# Enhanced dehydroepiandrosterone levels are positively correlated with N3 sleep stage in long-term mindfulness meditation practitioners

**DOI:** 10.5935/1984-0063.20220039

**Published:** 2022

**Authors:** Ravindra P. Nagendra, Talakad N. Sathyaprabha, Bindu M. Kutty

**Affiliations:** Center for Consciousness studies, Department of Neurophysiology National Institute of Mental health and Neurosciences (NIMHANS), India.

**Keywords:** Mindfulness, Sleep, Dehydroepiandrosterone, Hydrocortisone, Melatonin, Sleep, Slow-Wave

## Abstract

**Objectives:**

Meditation practices positively influence the neural, hormonal and autonomic systems. We have demonstrated that long-term practice of mindfulness meditation increases N3 and rapid eye movement (REM) sleep stages and bring efficient autonomic modulation during sleep. In the present study, the probable humoral correlation that could bring about these changes is evaluated.

**Material and Methods:**

Long-term Vipassana meditators (n=41) and controls (n=24) (males, 30-60 years of age) underwent a two-day consecutive whole night polysomnography recording. During the second day, with exposure to 100Lux brightness, blood was sampled from the antecubital vein between 8-9 PM and in subsequent early morning. Sleep stage was scored as per American Society of Sleep Medicine (ASSM) guidelines for the second-day recording. Sleep-related hormones were estimated - melatonin by radioimmunoassay; dehydroepiandrosterone (DHEA), cortisol, growth hormone (GH) and prolactin with enzyme-linked immunosorbent assay (ELISA); DHEA/cortisol ratio was calculated. Percentage of sleep stages and hormonal levels were compared between both groups using independent ‘t’ test and Pearson’s correlation was estimated between sleep stages and hormonal levels.

**Results:**

Meditators showed increased N3, REM sleep stages. Though evening cortisol was comparable between the two groups; early morning cortisol, diurnal DHEA and melatonin were significantly higher in meditators. Diurnal DHEA correlated significantly with the N3 sleep stage in meditators.

**Discussion:**

Higher diurnal DHEA despite variations in corresponding cortisol in meditators demonstrates that long-term Vipassana meditation practice modulates the hypothalamicpituitary-adrenal (HPA) axis and thereby influences sleep. Thus, the study provides evidence to explore the mechanism most likely involved with mindfulness meditation intervention in insomnia.

## INTRODUCTION

Sleep quality is one of the important determining components associated with health and well-being^1^. Growing evidence demonstrates that mindfulness meditation practice modulates various psycho-physiological processes and moderately facilitate proper sleep. These effects are reported in both general and clinical populations^2-4^. Most of the studies evaluate sleep either by questionnaire, sleep diary or actigraphy^5-8^, however, the whole night polysomnography is the gold standard for evaluation of sleep architecture.

The foremost polysomnography evaluation on the effect of meditation on sleep was conducted among transcendental meditation (TM) practitioners^9^ and showed that the senior TM practitioners have higher slow-wave sleep and rapid eye movement (REM) sleep when compared to the age-matched nonmeditating controls. Further, during slow-wave sleep, meditators showed higher theta-alpha power, which the authors ascribe to a higher state of consciousness during sleep. We have reported the differential effect of breath-based meditation practice (Sudarshan kriya yoga) and mindfulness meditation practice (Vipassana meditation) on sleep architecture. The former enhances slowwave sleep but has no effect on REM sleep; whereas the latter increases both slow-wave sleep and REM sleep even in the older age group and enhances REM density^10-12^. Despite attenuation of slow-wave generating mechanism with the normal ageing process^13^, our observation of retaining slow-wave sleep even in elderly long-term Vipassana meditation practitioners^11^ is attributed to the meditation-induced neural plasticity changes. Effective thalamocortical and corticocortical interactions are reported among meditators, which facilitates the generation of slow waves^14^. Even short-term Vipassana meditation practice is reported to enhance slow frequency oscillations during NREM sleep, implying neural plasticity changes^15^. Further, effective buffering of overt sympathetic surges during sleep is observed in long-term Vipassana meditation practitioners that could also facilitate a stable sleep^16^.

In addition to the neural and autonomic influences, sleep is also modulated by the hormonal system. Meditation practices induce both immediate and long-term effects on the hormonal milieu. In general, the practice of meditation reduces cortisol and catecholamines^17,18^, increases dehydroepiandrosterone (DHEA), melatonin, growth hormone (GH), thyroid-stimulating hormone (TSH) and prolactin^19-24^. Among various hormones, DHEA, cortisol and melatonin are considered as a metric to assess the effect of meditation practice on the humoral system^25^ and these hormones are also involved in the regulation of sleep^26^. An increase in melatonin levels^23,24,27,28^ and reduction in the cortisol response to a stressor^17,22,29,30^ is reported immediately after a meditation session. However, with the long-term practice of mindfulness meditation, enhanced melatonin and reduced cortisol are observed at the basal level^31-33^. On the contrary, long-term practice of TM is known to increase DHEA; reduce prolactin, growth hormone and TSH, without much changes in cortisol levels^19,21,34^. Mindfulness meditation intervention in cancer patients improves sleep quality and is associated with reduced random cortisol levels without affecting melatonin and DHEA^25^. But higher early morning cortisol levels is attributed to adequate sleep and is observed in yoga and mindfulness practitioners but not with TM practice^35-37^. These inconsistencies on the effect of meditation practices on hormones are not yet resolved though it was first reported decades back^38^, and the influence of meditation practice on sleep-associated hormonal profiles is sparingly evaluated. Our earlier studies have shown that long-term Vipassana meditation practice, by inducing neural plasticity changes and modulating cardiac autonomic activity will positively influence sleep architecture^16,39^. In the present manuscript, we have evaluated sleep-related hormones and their correlation with sleep stages among long-term practitioners of Vipassana meditation.

## MATERIAL AND METHODS

The data for the study were from a subgroup of participants from our earlier report comprising of 91 subjects (mindfulness meditators and controls) who underwent consecutive two whole night polysomnography recordings^11^. Out of 91 subjects, 65 of them gave written consent for hormonal assay along with whole night polysomnography recordings. The sleep data and hormonal assessment of these subjects, i.e., mindfulness meditators (n=41) and non-meditating controls (n=24) are being reported here. The study was initiated after obtaining approval from the Institute Human Ethics Committee (NIMHANS/XXXVII/IEC/2005).

The meditators were practising Vipassana meditation regularly for more than three years, daily for 2-4 hours, i.e.,1-2 hours, in the morning (between 6-8 A.M.) and 1-2 hours in the evening (between 6-8 P.M.). The details of meditation training have been elaborated on in our earlier publication^11^. In brief, Vipassana meditation is possibly the most ancient form of mindfulness-based meditative technique. Presently the technique follows the tradition of Sayagyi U Ba Khin as taught by S. N. Goenka all over the globe. The word ‘Vipassana’ in the Pali language means ‘to see things as they are’. Vipassana meditation practice involves the strategy of mindfulness wherein, the meditators learn to notice and witness the perceptions of the senses and the thoughts arising in the mind without reacting to them, like an onlooker, and to focus their attention on their bodily activities in their true perspective, in their true nature. The Vipassana meditators in the present study were recruited through the Vipassana Research Institute at Igathpuri, India, the world headquarters for Vipassana meditation. All the meditators were employed in various private and public sector institutions and leading normal social life, none of them were monks nor lived in a monastery. The control subjects were recruited from various private and public sector institutions. Participants who were practising any other form of meditation, yoga, or regular physical activity were excluded. Subjects from both groups who consumed tobacco, alcohol, and regular medications that could potentially influence sleep and hormonal profile were excluded. Subjects from both groups were matched for demographic characteristics such as age, socioeconomic and educational status. Before recruitment, the clinical history of participants was obtained; their routine sleep habits were assessed and were examined for medical, psychiatric, neurological and other disease conditions. Participants maintained a sleep log for a week before the study. Those with a history of sleep disorders for three months and beyond, sleep-log showing sleep disturbance/ restriction or deprivation, on regular medications for neuropsychiatric conditions were excluded from the study.

Participants were housed in the institute guesthouse with a uniform diet. They were instructed to refrain from the consumption of coffee or tea after 4 p.m. and asked to report to the Human Sleep Research Laboratory after their dinner. With all the precautions, 5ml of blood from the antecubital vein was drawn between 8-9 P.M. before sleep recordings. Subjects woke up the next morning without an alarm and after half an hour the blood was drawn. Blood was collected in uniform conditions with the subject being seated and the room being illumined with the intensity of about 100Lux. Blood samples were immediately centrifuged at 3000rpm for 15min and serum was separated, aliquot in 5ml vials, and stored at-800ºC.

The whole night polysomnography was recorded using a 32-channel digital EEG system (EEG- 2110, Nihon Kohden Corporation, Japan) with EEG, EOG, and chin EMG electrodes. The technical details are elaborated elsewhere^11^. The recording was carried out until the subjects woke spontaneously by themselves in the morning. The offline scoring of sleep was carried with ASSM criteria^40^. The sleep scoring was carried independently by two experienced sleep scorers and the reliability of scoring from both of them was about 95.04%. Sleep efficiency index (SEI) was calculated using the formula (TST + TIB) x100, where, TST is total sleep time and TIB is the total time in bed. The recordings which had SEI of more than 85% were selected for the analysis. SEI based selection ensures the exclusion of possible sleep-related problems so that the analysis is carried out only among good sleepers.

Melatonin was estimated by radioimmunoassay (RIA)^41^. Radioimmunoassay (Biosource Europe SA, Belgium) involves competition between a radioactive and non-radioactive antigen for a fixed number of antibody binding sites. The amount of I125 labelled antigen bound to the antibody is inversely proportional to the analyte concentration of the sample. When the system is in equilibrium, the antibody-bound radioactivity is precipitated with a second antibody in the presence of polyethylene glycol. The precipitate is counted using a gamma counter. Quantification is achieved by comparing their activity with a reference curve prepared with a known standard. The intra-assay coefficient of variation of melatonin is 12.3%.

DHEA, cortisol, GH, and prolactin were analyzed by enzyme-linked chemiluminescence (ELISA) (Immulite of Siemens Medical Solutions). The general principle of ELISA is applied for the assay of hormones, wherein the hormone specific antibodies are used. Beads coated with the specific hormone antibody are mixed with a reagent containing alkaline phosphatase, conjugated with a rabbit antibody. Reagent with serum is incubated with antibody-coated beads. Antigen (hormone) in the serum forms a complex with antibody sandwitched with the reagent. The unbound enzyme is removed by centrifugal wash and a chemiluminescence’s substrate is added to the bead. The signal is generated in proportion to the bound enzyme. Intra-assay coefficient variation for prolactin and DHEA is 9.5%, GH is 5.3%, and cortisol is 8.8%. Subsequently, the DHEA/cortisol ratio was calculated.

### Statistics

The statistical test was applied using SPSS version 24. Descriptive statistics of sleep variables (mean and standard deviation) and hormonal profile (mean and standard error) were calculated. The hormonal levels were positively skewed (skewness >2); therefore, the values were log-transformed for statistical analysis. However, in the results, the original values are depicted.

Independent ‘t’ test was used to compare the percentage of sleep stages and logarithmic values of hormonal profile between the two groups. A paired ‘t’ test was used to compare the diurnal differences in logarithmic hormonal levels within controls and meditators. The correlation was estimated between various sleep stages and hormonal profiles using Pearson’s correlation. And *p*<0.05 is the level of significance.

## RESULTS

Both the groups were comparable for their age [controls = 44.67±9.72 years, meditators = 45.04±9.56 years, t=0.63, *p*=0.52]. No significant difference in the duration of sleep is observed between the two groups [controls = 357.14±63.36 minutes, meditators = 375.98±62.07 minutes, t=1.30, *p*=0.25]. Sleep efficiency index even though was above 90% in both, meditators showed significantly higher value [controls = 91.85±4.19 percentage, meditators = 93.98±3.43 percentage, t=2.1, *p*=0.036]. Whereas microarousal index during sleep was comparable between two groups [controls = 15.26±8.01, meditators = 16.42±8.01, t=0.41, *p*=0.68].

**Figure 1:**
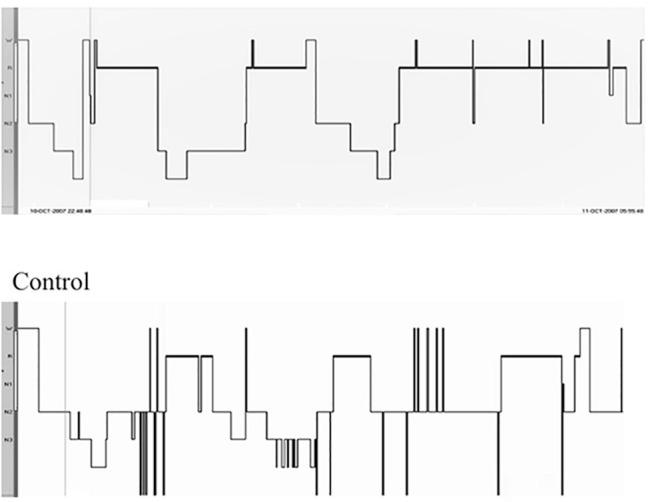
Representative hypnogram of meditator and control subject.

Notes: Representative hypnogram of meditator and control; meditator shows enhanced N3 and REM sleep state than control.

In meditators, the percentage of sleep stage N1 [controls = 19.29±11.18, meditators = 12.00±8.55, t=2.85, *p*=0.006] and N2 [controls = 50.43±11.55, meditators = 42.09±11.75, t=2.71, *p*=0.009] was less, whereas, percentage of N3 [controls = 6.86±1.39, meditators = 15.57±9.06, t=4.01, *p*=0.000] and REM sleep stages [controls = 22.40±7.76, meditators = 32.20±8.89, t=4.39, *p*=0.000] were significantly higher when compared to controls. The representative hypnogram of a meditator and control is given in Figure 1.

**Figure 2:**
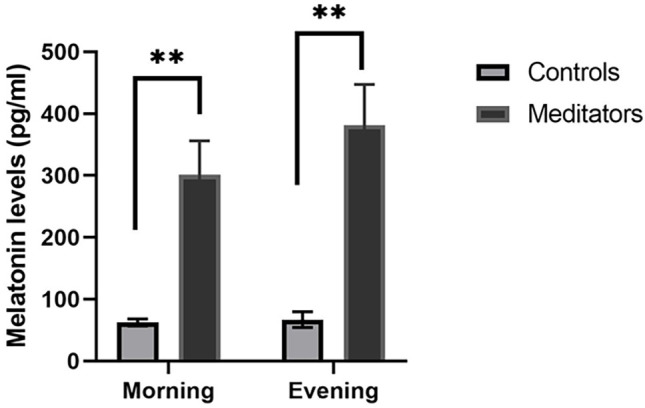
Comparison of morning and evening levels of melatonin between controls and meditators.

Notes: Melatonin levels, both during morning and evening are significantly high in meditators than controls; **p<0.001.

The details of the hormonal levels are provided in Table 1. Diurnal melatonin levels were significantly higher in meditators than controls [morning t=4.05, *p*=0.00, evening t=4.05, *p*=0.00] (Figure 2), but, did not show any significant differences within controls and meditators. Similarly, DHEA levels both during morning and in the evening were higher among meditators than controls [morning t=3.16, *p*=0.04, evening t=3.08, *p*=0.04] without significant diurnal variations within each group. Cortisol levels in meditators, when compared to controls, showed higher levels in morning sample [t=2.04, *p*=0.04], but, evening levels was comparable [t=0.37, *p*=0.70]. Whereas, significantly higher evening cortisol levels were observed in both controls and meditators when compared to their own morning levels. Both GH [morning t=1.38, *p*=0.19, evening t=0.09, *p*=0.56] and prolactin [morning t=0.08, *p*=0.99, evening t=1.62, *p*=0.20] did not show any significant difference between the two groups. Diurnal variations of prolactin in both controls [t=3.34, *p*=0.003] and meditators [t=4.38, *p*=0.00] showed increased levels in the evening, however, neither of the groups showed any diurnal difference in GH levels. DHEA/ cortisol ratio was comparable between the groups [morning= t=0.45, *p*=0.65, evening t=0.96, *p*=0.33] and the ratio was significantly less in the evening in both controls and meditators.

**Table 1:** Comparison of diurnal levels of melatonin, DHEA, cortisol, GH, prolactin, and DHEA/cortisol ratio between controls and meditators.

		Control (n=24)	Meditators (n=41)	*p*-value
Melatonin (pg/mL)	Morning	62.95±5.69	301.53±54.44	<0.001
	Evening	66.95±12.65	381.80±65.51	<0.001
		t=2.8, *p*=0.001	t=1.28, *p*=0.21	
DHEA (pg/dL)	Morning	169.39±36.23	192.37±24.21	0.04
	Evening	156.72±36.22	213.25±33.14	0.04
		t=0.83, *p*=0.041	t=1.38, *p*=0.18	
Cortisol (Pg/dL)	Morning	7.52±0.88	10.26±0.90	0.04
	Evening	15.31±1.02	15.85±0.93	0.70
		t=4.83, *p*=0.00	t=3.54, *p*=0.001	
Growth hormone (ng/dL)	Morning Evening	0.14±0.05 0.33±0.70	0.30±0.10 0.22±0.33	0.19 0.56
		t=1.41, *p*=0.17	t=0.57, *p*=0.51	
Prolactin (ng/dL)	Morning	12.93±1.52	12.95±1.76	0.99
	Evening	23.94±12.95	19.51±1.96	0.20
		t=3.34, *p*=0.003	t=4.38, *p*=0.00	
DHEA/Cortisol ratio	Morning	28.06±5.55	24.82±4.41	0.65
	Evening	10.73±1.93	13.80±2.21	0.33
		t=3.17, *p*=0.005	t=2.26, *p*=0.02	

Notes: Meditators show significantly higher diurnal levels of melatonin, DHEA, and morning levels of cortisol when compared to controls. Evening levels of melatonin, cortisol, and prolactin were higher in controls when compared to their morning levels. Meditators showed a significantly higher evening cortisol and prolactin levels than their morning levels. Values in mean ± SE, *p*<0.05 as significant level.

**Figure 3:**
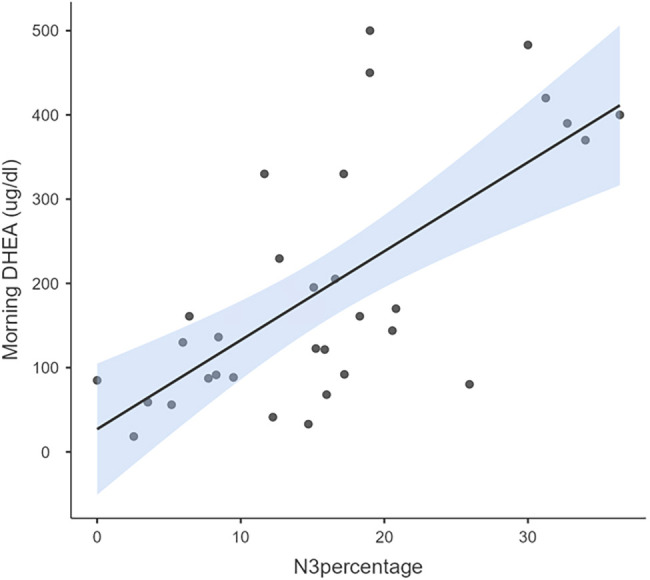
Correlation between morning DHEA levels and N3 percentage in meditators.

Notes: Morning DHEA levels correlated significantly (r=3.16, *p*=0.04) with the percentage of N3 in meditators.

**Figure 4:**
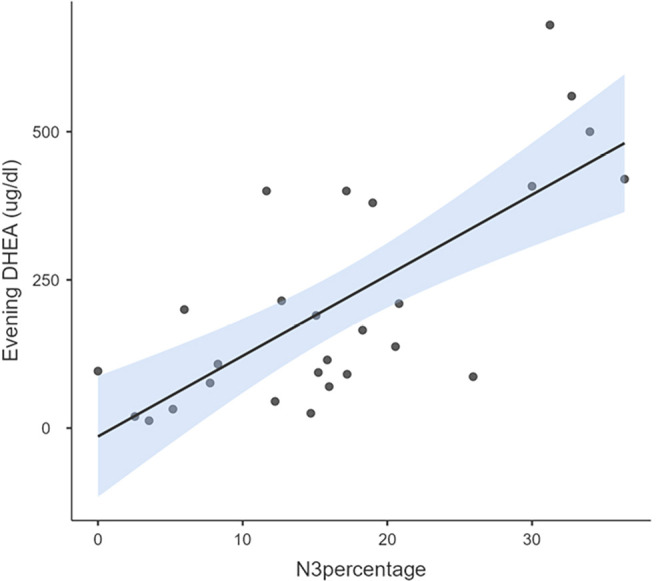
Correlation between evening DHEA levels and N3 percentage among meditators.

Notes: Evening DHEA levels correlated significantly (r=3.08, *p*=0.04) with the percentage of N3 in meditators.

Average years of meditation practice by meditators was 7.29±2.32 years of practice. The Pearson’s correlation with meditation duration showed a positive relation with levels of DHEA (morning r=0.36, *p*=0.03), prolactin (morning r=0.73, *p*=0.00, evening r =0.52, *p*=0.002) and DHEA/cortisol ratio (morning r=0.41, *p*=0.02). Sleep stages in meditators showed a significant positive correlation between stage N3 with diurnal DHEA [morning r=0.68, *p*=0.00, evening r=0.72, *p*=0.00, (Figures 3 and 4)], morning melatonin [morning r=0.42, *p*=0.02] and DHEA/cortisol ratio [morning r=0.58, *p*=0.00]. Otherwise, no significant correlation was found with percentage of N1, N2 and REM sleep. Among controls, only morning cortisol levels showed significant negative correlation with percentage of N2 sleep stage (r=-0.58, *p*=0.003).

## DISCUSSION

The present study assessed the levels of sleep-related hormonal profiles and their correlation with sleep stages among long-term practitioners of Vipassana meditation. Vipassana meditators showed significantly higher diurnal levels of DHEA, melatonin and morning cortisol levels, besides increased N3 sleep stage demonstrating a moderate to strong significant positive correlation with diurnal DHEA levels. There are consistent reports demonstrating a positive effect of meditation practice on subjective sleep quality despite the variability in its correlation with hormonal profiles^2,38^. However, to the best of our knowledge, this could be the first study to report the relationship between sleep-related hormones with whole night polysomnographic data among long-term Vipassana meditation practitioners.

Meditation practice is known to increase DHEA in men (45-70 years) and women (20-74 years) demonstrating the robust effect even after adjusting for other confounding variables like diet, body mass index, and physical activity^19^. Increased diurnal DHEA levels in the middle-aged male meditators corroborate with this report. A healthier shift of DHEA is observed in patients who practised mindfulness meditation and yoga for 2 months; however, mindfulness practice also showed improvement in sleep quality^25,42^. In contrast, three years of yoga practice showed improvement in sleep quality, without any changes in DHEA^43^. Thus, reinforcing the observation that type and duration of meditation practice have a differential effect on hormonal profiles^44^. In the present study, long-term practitioners of Vipassana meditation have shown high levels of diurnal DHEA with a significant positive correlation with N3 sleep stage. Lower DHEA levels, in healthy individuals, is known to induce sleep disturbance and reduces slow-wave sleep in patients with obstructive sleep apnea^45,46^. However, few studies have observed no significant relationship between DHEA levels either with sleep duration and other polysomnography variables^47,48^. These contrasting observations could be due to the influence of DHEA on multiple pathways associated with sleep-promoting or inhibiting mechanisms^49^. These mechanisms may include DHEA-driven variations in testosterone and estradiol secretion which promotes or inhibits sleep respectively^50^ or influencing agonistic and antagonistic action on GABAergic activity which influences NREM and REM sleep^49,51^. Therefore, DHEA at appropriate dosage is considered as supplementation therapy for various sleep disorders^52^. Further, DHEA is known to have an antiaging effect^53,54^ and the observation of a positive correlation between DHEA and N3 sleep stage among meditators in the present study is in accordance with our earlier finding of mitigating age-associated changes in sleep architecture among long-term Vipassana meditation practitioners^11^.

In the present study, meditators, when compared to controls, showed a higher level of cortisol in the morning. Whereas, evening cortisol levels were comparable between them. Our observation of higher cortisol levels in the morning appears to be counterintuitive where meditation practice is expected to decrease the cortisol levels^18^. Though higher morning cortisol levels, was reported to be proportional to perceived stress^55^, studies consistently have demonstrated a blunted response to chronic stress conditions in apparently healthy individuals^56,57^ and also in various medical and psychiatric conditions^58^. The blunted response is attributed to the reduced sensitivity of adrenals to circulating adrenocorticotropin (ACTH) hormone which is regulated by the hypothalamic-pituitary-adrenal (HPA) axis^59,60^. Therefore, impaired early morning cortisol is considered to be the marker of alteration in the function of the HPA axis due to prolonged stress^61^.

The most accepted functional role of early morning cortisol is that it serves as an allostatic boost on awakening to prime the neural network to modulate cognition anticipating the demands of the day^62^ and facilitates wakefulness with better resilience^59,63^. This allostatic boost appears to be more in the early morning riser^64,65^ irrespective of the mode of waking up, i.e., spontaneously or by an alarm^66^ and chronotype of the person^67^. There are reports showing higher morning cortisol levels with mindfulness meditation practice^36,68^. However, the latest report on TM practice in young adults shows no increase in early morning cortisol levels when compared to the waitlist control group^37^. It could be attributed that type and duration of meditation practice will differently influence HPA activity. In response to cortisol, the sensitive HPA axis secretes DHEA to mitigate the adverse actions of cortisol^69^. This protective response is reduced with prolonged stress^70^. In the present study, meditators showed higher diurnal DHEA levels. It is to be noted that though evening cortisol levels were comparable between both groups, meditators showed higher DHEA levels. Evening cortisol reflects the stress levels of the day^71^. This demonstrates that the HPA axis in meditators is more sensitive than controls. Our observation of higher DHEA levels in meditators and its correlation with N3 sleep stage provides a clue that the long-term Vipassana meditation practice could possibly influence the HPA axis to be more resilient and dynamic, and favourably influence sleep. However, the cortisol response to early morning wake is known to vary on situational factors like prior day experiences, the anticipation of the day ahead and its related stress and changes in weather/seasonal conditions^58,71^. More studies are warranted in this direction accounting for other factors influencing morning cortisol levels.

Melatonin is the most versatile hormone preserved through evolution^72^. In the present study, we observed significantly higher diurnal melatonin levels in meditators and N3 sleep stage showed a positive correlation with morning melatonin levels. Whereas, among controls, no correlation was observed between melatonin levels and sleep stages. Various types of meditation practices like mindfulness, TM, Omkar meditation has been shown to increase nocturnal melatonin levels (both saliva and plasma) immediately after a meditation session and high salivary levels are maintained for long hours after meditation^23,24,27,28^. It is to be noted that long-term meditators have shown higher basal melatonin^73^, thus, showing the duration of meditation practice differently influence melatonin. Further, melatonin levels depend on the type of biological specimen and methodology used to estimate. Melatonin in plasma is ten times more than in the other biological specimens. In the present study, plasma levels of melatonin were estimated using a more sensitive method the radioimmunoassay with I125 labelled iodomelatonin^41^. Other confounding factors that can influence melatonin levels like circadian variations, exposure to light and diet were relatively taken care of. All participants were housed in the guesthouse with a uniform diet and blood was drawn during the same clock hours exposed to 100Lux brightness in the laboratory. Both controls and meditators were with their normal social and professional responsibilities with no shift work. This demonstrates that circadian phase shifts were not present in the participants, which otherwise would have been a major confound. As per the norms^73^, the melatonin levels were within the normal range in controls and acceptable maximal upper limit in meditators.

Melatonin administration in normal healthy volunteers and in various clinical conditions is known to improve sleep by reducing sleep onset latency, increasing sleep duration and efficiency^74-78^. On the contrary, there are reports showing no effect^79^ or influences only by reducing the sleep onset latency^80^. Similarly, variable effects of melatonin on sleep stages are reported. Studies have shown reduced slow-wave sleep with either decrease^81^ and increase^82^ of sleep stage N2 with enhanced^81^ or no changes^82^ in REM sleep. Few have shown no effects of melatonin on sleep stages^83-86^ except one study demonstrating only reduction in REM onset latency^87^. The reasons for inconsistent results could be due to variations in the dosage, timing and mode of administration. Further, the methodology used to acquire sleep data could also influence the outcome, where variations in the quantification of few sleep variables are reported between actigraphy and polysomnography^88^. In the present study, polysomnographically (gold standard method) scored N3 sleep stage in meditators showed a positive correlation with morning melatonin levels. Whereas, controls did not show any correlation between their melatonin level and sleep stages. The reason for such observation cannot be deciphered from the present protocol. It is recommended to have a serial blood sampling throughout the night for melatonin estimation to capture the circadian variations along with other physiological variables like temperature for a deeper insight. However, it is important to mention that meditation practice significantly increases melatonin levels. Three reasons have been hypothesized for enhanced melatonin levels in meditators viz., reduced hepatic metabolism of melatonin or meditationinduced higher serotonin and noradrenaline levels, which are essential for melatonin synthesis^89^ or meditation-induced heightened activity of the pineal gland itself^90^. In the present study, meditation was not preceded before sleep nor prior to blood collection in the morning. Therefore, the immediate effect of meditation on hormone level is ruled out. Hence, high circulating melatonin levels observed in the study could possibly be a trait effect of intense meditation practice.

Melatonin is known to synergistically influence DHEA and enhances the inhibitory effect of GABA to improve sleep^91^. There is evidence to demonstrate the increased production of GABA in the brain with meditation practice^92-96^. With this background, we speculate that meditation-induced effects like higher levels of DHEA, melatonin and enhanced inhibitory effect of GABA could cumulatively have facilitated the slow-wave generation thereby enhancing N3 sleep stage. In addition, DHEA and melatonin are shown to have anxiolytic, antioxidant, anti-stress, antiaging, immuno-modulation and other hosts of physiological and psychological benefits^21,73-75^ could have also influenced sleep in meditators. More studies are warranted especially in assessing the circadian variations of hormones with serial sampling and estimating GABA levels to establish the causal relationship between hormonal levels and sleep architecture in meditators. Other hormones like growth hormone and prolactin did not show any correlation with sleep stages in both controls and meditators.

In conclusion, long-term Vipassana meditation practice increases diurnal DHEA, melatonin and morning cortisol levels. Though all three hormones are known to influence sleep, we observed a positive correlation of N3 sleep stage only with diurnal DHEA. An increase in DHEA with cortisol levels in meditators reflects the positive influence of meditation practice on the HPA axis. This humoral correlate with sleep architecture is in addition to the neural plasticity and autonomic modulatory effects of Vipassana meditation practice on sleep^11,16,39^. This study provides ample evidence to explore the mechanisms that probably could have been involved in the beneficial effect of mindfulness meditation intervention in insomnia. However, our observation is limited to long-term practitioners of Vipassana meditation. The circadian variations of these hormones and other related factors like core body temperature were not estimated in the study. To get more insight further experiments are warranted with varying duration of Vipassana meditation practice and estimating the circadian variations of hormones.
